# Lung ultrasonography findings of coronavirus disease 2019 patients: Comparison between primary and secondary regions of China

**DOI:** 10.1002/iid3.713

**Published:** 2022-09-27

**Authors:** Jing Han, Xi Yang, Wei Xu, Ronghua Jin, Sha Meng, Lei Ding, Yuan Zhang, Xing Hu, Weiyuan Liu, Haowen Li, Fankun Meng

**Affiliations:** ^1^ Ultrasound and Functional Diagnosis Center, Beijing You An Hospital Capital Medical University Beijing China; ^2^ Department of ultrasound Hanyang Hospital Affiliated to Wuhan University of Science and Technology Wuhan China; ^3^ Key Laboratory of Carcinogenesis and Translational Research (Ministry of Education/Beijing), Department of Hepato‐Pancreato‐Biliary Surgery Peking University Cancer Hospital and Institute Beijing China; ^4^ Beijing You An Hospital Capital Medical University Beijing China; ^5^ Department of Science and Technology, Beijing You An Hospital Capital Medical University Beijing China; ^6^ Ultrasonography, China Aerospace Science and Industry Corporation 731 Hospital Beijing China

**Keywords:** coronavirus disease 2019, lung ultrasound score, ultrasonographic features, ultrasonography

## Abstract

**Background:**

An unexplained pneumonia occurred in Wuhan, China in December 2019, later identified and named coronavirus disease 2019 (COVID‐19). This study aimed to compare the ultrasonographic features of the lung between patients with COVID‐19 in Wuhan (the primary region) and those in Beijing (the secondary region) and to find the value of applying ultrasound in COVID‐19.

**Methods:**

A total of 248 COVID‐19 cases were collected, including long‐term residents in Wuhan (138), those who had a short‐term stay in Wuhan (72), and those who had never visited Wuhan (38). Ultrasound examination was performed daily; the highest lung ultrasound score (LUS) was the first comparison point, while the LUS of the fifth day thereafter was the second comparison point. The differences between overall treatment and ultrasonography of left and right lungs among groups were compared.

**Results:**

The severity decreased significantly after treatment. The scores of the groups with long‐term residence and short‐term stay in Wuhan were higher than those of the group that had never been to Wuhan.

**Conclusion:**

Ultrasonography is effective for dynamic monitoring of COVID‐19. The ultrasonographic features of patients in the Wuhan area indicated relatively severe disease. Thus, Wuhan was the main affected area of china.

## Graphical abstract

Ultrasonography is effective for monitoring coronavirus disease 2019 in lung lesions. The lung lesions in the main affected area were serious.

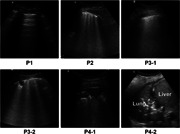

## 1 INTRODUCTION

In December 2019, patients in Wuhan City, Hubei Province, China, were diagnosed with novel coronavirus pneumonia.^1^ The International Committee on Taxonomy of Viruses named this severe acute respiratory syndrome coronavirus‐2 (SARS‐CoV‐2). This virus can cause a severe respiratory disease similar to that caused by severe acute respiratory syndrome (SARS) and Middle Eastern Respiratory Syndrome (MERS) coronaviruses. This disease was named novel coronavirus pneumonia. On February 11, 2020, World Health Organization (WHO) named the disease caused by infection with novel coronavirus as coronavirus disease 2019 (COVID‐19).^2^ It has spread rapidly not only in China but also worldwide,^3^ and has become a public health emergency of international concern, with COVID‐19 infection posing a major threat to global health.

At present, diagnosis is mainly carried out by reverse transcription‐polymerase chain reaction (RT‐PCR) and chest high‐resolution computed tomography (CT); ultrasound is also becoming more commonly used in diagnosis and treatment. Ultrasound imaging has clinical diagnostic value for lung diseases, especially peripheral lung diseases. We aimed to further investigate the value of ultrasound imaging for COVID‐19 diagnosis by analyzing differences in ultrasound diagnosis among different regions and determining whether differences exist in the pathological degrees of patients in different regions. We found that the clinical symptoms of patients in primary areas are generally more severe than those of patients in secondary areas. Currently, no literature exists comparing the ultrasonic images of patients in different areas. According to China's COVID‐19 policy, all patients with confirmed diagnoses must be hospitalized for observation and treatment. This policy provides a reliable basis for the comparison of patients' conditions between the two regions. In this study, 248 COVID‐19 cases in Wuhan and Beijing were scored for pulmonary lesions to understand the similarities and differences between patients in Wuhan and Beijing, allowing a more comprehensive understanding of COVID‐19 and facilitating improved treatment.

## 2 MATERIALS AND METHODS

### 2.1 Study patients

This was a cross‐sectional observational study. Wuhan was regarded as the primary area because it was the first city affected by COVID‐19 in China, with a fast transmission rate, a rapid increase in the number of infected patients, and a serious overall condition. Cases appeared in the other cities subsequently, and there the overall condition was relatively mild; thus, we wanted to determine whether there were also different manifestations in the ultrasound imaging.

This work was supported by the Ministry of Science and Technology of the People's Republic of China (grant number 2020YFC0841700). The study was approved by the Ethics Committee of Beijing You An Hospital, affiliated with the Capital Medical University ([2020]020). Written informed consent was signed by all patients. We enrolled 138 and 110 patients (72 cases with short‐term stay in Wuhan, and 38 cases with never visited Wuhan) from the Wuhan Hanyang Hospital and the Beijing You An Hospital, respectively, who were diagnosed with COVID‐19 (all hospitalized patients in the same period were included, except those meeting the exclusion criteria). Both hospitals were designated hospitals for COVID‐19 patients. They were divided into three groups, comprising those with long‐term residence (more than 3 months) in Wuhan (138 cases), those who had a short‐term stay (within 3 months) in Wuhan (72 cases), and those who had never visited Wuhan (38 cases). Ultrasonographic examination of the lungs was performed every day from admission, and the scores were assigned according to the unilateral six‐zone protocol of lung ultrasonography. The highest lung ultrasound score (LUS) was considered the first comparison point, while that on the fifth day thereafter was considered as the second comparison point. The overall treatment status, the differences in scores among different groups of patients, and involvement of the left and right lungs was compared. Patients with a history of chronic lung disease and those with no lesions on CT images or those with lesions that had not spread to the periphery of the lung were excluded from the study. The patient's age, sex, and contact history were recorded. The presence of other symptoms, such as fever, cough, diarrhea, and other related symptoms, were recorded. In addition, some laboratory‐related indicators were collected from patients who had symptoms and had been admitted to the hospital within 3 days of onset of symptoms. The laboratory results were collected on the third day after symptoms first appeared.

### 2.2 Lung ultrasound

**Figure 1:**
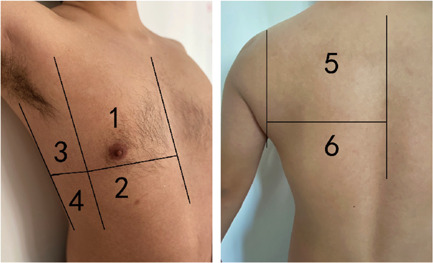
Lung ultrasound score

**Figure 2:**
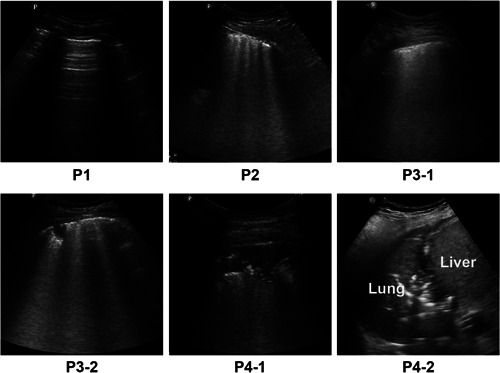
Four types of ultrasound scoring features, P1 normal ventilation (N‐type): the ultrasonographic features showed A‐line sign or ≤2 independent B lines, indicating good lung inflation. P2 (B1‐type): Multiple B lines, with an interval of about 7 mm between the B lines (B7 lines). P3‐1 and P3‐2 (B2‐type): Multiple B lines, with an interval ≤3 mm between the B lines (B3 lines), with or without small patchy consolidation. P4‐1 and P4‐2 (C‐type): The ultrasonographic features were hepatization or fragment sign of lung tissue, with dynamic bronchial inflation sign, with or without a small amount of pleural effusion, indicating consolidation of the lung. N = 0, B1 = 1, B2 = 2, C = 3.

Caltabeloti and Rouby^4^ proposed division of a unilateral lung into six zones for examination. With the anterior and posterior axillary lines as the boundaries, the lung was divided into three zones, namely, the anterior, lateral, and posterior zones (Figure 1). Each zone was further divided into upper and lower parts. The worst sign observed in each zone during the examination was considered as the final judgment of the zone, and the results were recorded by classification into the following four basic types (Figure 2): type N, the ultrasonographic features showed an A line or ≤2 independent B lines, indicating good lung inflation; type B1, the ultrasonographic features showed multiple B lines, with an interval of about 7 mm between the B lines (B7 lines); type B2, the ultrasonographic features showed multiple B lines, and the interval between B lines was ≤3 mm, (B3 lines); and type C, the ultrasonographic features showed hepatization or fragmentation of the lung tissue, with dynamic bronchial inflation, with or without a small amount of pleural effusion, indicating consolidation of the lung. The scoring of lung ultrasonography was based on the following four types: N = 0, B1 = 1, B2 = 2, and C = 3. Meanwhile, we also considered the shortcomings of these scoring criteria, outlined in the guidelines proposed by Lichtenstein^5^ to conduct a comprehensive evaluation (e.g., for P3‐2 in Figure 2, although there was consolidation, the scope was very small; thus, we did not assign 3 points but 2 instead). All patients underwent ultrasonographic lung examination, and the sum of the scores of the 12 zones was recorded. All lung ultrasound images were performed by two experienced sonologists.

### 2.3 Statistical analysis

SPSS (version 26.0) was used for statistical analyses. The measurement data satisfying normal distribution were expressed as mean ± standard deviation (mean ± SD). Data not satisfying normal distribution were expressed as median (range). The one‐way analysis of variance was used for ages that satisfied a normal distribution. For other measurement data that did not satisfy a normal distribution, a nonparametric test of independent samples and related samples was used. Wilcoxon's test was used for intragroup before‐versus‐after comparison, and the Mann–Whitney *U* test was used for comparisons between two independent samples. The *χ* ^2^ test was used for the comparison of rates. *p* < 0.05 were considered statistically significant.

## 3 RESULTS

### 3.1 Baseline information

Among the 248 patients, 132 were males and 116 were females, with an average age of 50 ± 11 years. They were divided into the following groups: patients who had long‐term residences in Wuhan, those who had a short‐term stay in Wuhan, and those who had never visited Wuhan. The sex, age, and presence of fever, cough, myalgia, diarrhea, and related clinical characteristics of the three groups are shown in Table 1.

**Table 1:** General characteristics of patients

Characteristics	Long‐term residence in Wuhan (*n* = 138)	Short‐term stay in Wuhan (*n* = 72)	Never visited Wuhan (*n* = 38)	*χ* ^2^/*F*	*p* Value
Sex (M/F)	71/67	40/32	21/17	0.395	.821
Age (years	51.1 ± 11.1	48.3 ± 11.8	49.6 ± 10.2	1.444	.238
Fever	115 (83.3%)	59 (81.9%)	33 (86.8%)	0.436	.804
Cough	83 (60.1％)	42 (58.3%)	21 (55.2%)	0.305	.858
Myalgia	20 (14.5%)	9 (12.5%)	6 (15.8%)	0.259	.879
Diarrhea	14 (10.1%)	9 (12.5%)	4 (10.5%)	0.277	.871
Leukopenia	29 (21.0%)	15 (20.8%)	7 (18.4%)	0.127	.938
Lymphopenia	109 (78.9%)	48 (66.7%)	24 (63.2%)	5.839	.054
Thrombocytopenia	47 (34.1%)	21 (29.2%)	10 (26.3%)	1.074	.581
CRP elevation	115 (83.3%)	53 (73.6%)	27 (71.1%)	4.194	.123
Lactic acid level	79 (57.2%)	32 (44.4%)	16 (42.1%)	4.592	.101

*Note*: Data are presented as either mean (SD) or no. (%), *p* < .05.

Abbreviation: CRP, C‐reactive protein; F, female; M, male.

### 3.2 LUS

The scores of the 248 patients decreased significantly by the fifth day after the highest overall scores. A significant difference was seen between the highest scores and those on the fifth day thereafter among the patients in the three groups. For the three groups with different left and right lung involvement statuses, significant differences were also seen between the highest scores and those obtained on the fifth day after treatment (Table 2). On the fifth day after that with the highest scores, the range of B3 lines had significantly reduced, and some patients showed B7 lines. The scope of the original B7 lines had also reduced and partially recovered to normal. B7 lines were detected in only a few areas, without any pathological changes. Local pathological changes were considered to have extended to the periphery of the lung lobes. Six of the 248 patients died owing to the worsening of the disease. The consolidation in both lungs gradually worsened, pleural effusion increased, and some patients were supported by extracorporeal membrane oxygenation because of respiratory failure. The other patients recovered (LUS and CT diagnosis) and were discharged.

**Table 2:** Highest scores among 248 patients, scores of each group obtained after 5 days, and scores of the left and right lungs

Groups	Gighest scores	After 5 days scores	*z*	*p* Value
All patients (*n* = 248)	12 (11,14)	9 (8,11)	−13.172	.000
Right lungs	6 (6,7)	4.5 (4,6)	−13.030	.000
Left lungs	6 (5,7)	4 (4,6)	−12.849	.000
Long‐term residence in Wuhan (*n* = 138)	13 (11,15)	9 (7,11)	−9.910	.000
Right lungs	7 (6,7)	4 (4,6)	−9.768	.000
Left lungs	6 (5,8)	4 (4,6)	−9.780	.000
All patients of Beijing (*n* = 110)	12 (11,14)	9 (8,11)	−8.687	.000
Right lungs	6 (5,7)	5 (4,5)	−8.700	.000
Left lungs	6 (5,7)	5 (4,6)	−8.384	.000
Short‐term stay in Wuhan (*n* = 72)	12 (11,14)	9 (8,11)	−6.929	.000
Right lungs	6 (6,7)	5 (4,5.75)	−6.929	.000
Left lungs	6 (5,7)	5 (4,6)	−6.847	.000
Never visited Wuhan (*n* = 38)	11 (10,13)	9 (8,11)	−5.381	.000
Right lungs	6 (5,6)	4 (4,5)	−5.372	.000
Left lungs	5.5 (5,7)	4.5 (4,6)	−4.976	.000

*Note*: Data are presented as median (p25, p75), *p* < .05.

The highest scores of the groups with long‐term residence in Wuhan and with a short‐term stay in Wuhan were higher than those of the group that had never been to Wuhan. There was no difference in the highest scores between the group with long‐term residence in Wuhan and the group that had a short‐term stay in Wuhan (Table 3).

**Table 3:** Differences in the highest scores among the three groups

Groups	Highest scores	*z*	*p* Value
Long‐term residence in Wuhan/short‐term stay in Wuhan	13 (11,15)/12 (11,14)	−1.813	.070
Long‐term residence in Wuhan/never visited Wuhan	13 (11,15)/11 (10,13)	−3.720	.000
Short‐term stay in Wuhan/never visited Wuhan	12 (11,14)/11 (10,13)	−2.197	.028

*Note*: Data are presented as median (p25, p75), *p* < .05.

## 4 DISCUSSION

Bedside lung ultrasound plays an important role in dynamic monitoring of COVID‐19 disease progression, and also greatly facilitates comparison of the severity of the disease in different areas of the lung. Our study found that ultrasonography can be used to directly monitor disease progression during the diagnosis and treatment of COVID‐19. We performed lung ultrasound examinations for hospitalized patients every day. Owing to the varying disease severities among patients at the time of admission, the ultrasound scores were quite different. Using the dynamic scores, we selected the highest score as the first time point and the score on the fifth day thereafter as the second time point and compared the score difference between the two points. We found that the score difference between the two points was significant. Excluding the six patients who died, the total number of B lines in the lungs was less than 5 at the time of discharge.^6^ This result is a strong affirmation of the value of ultrasonography. Besides RT‐PCR, CT has always been the gold standard for the diagnosis of lung diseases, and the diagnosis of COVID‐19 is no exception.^7^, ^8^, ^9^ However, because of the extremely poor penetrability of ultrasound through gases, ultrasonography is not ideal for the examination of lung diseases. Internal lesions can only be detected when lung water increases and spreads to surrounding areas or when consolidation occurs. However, ultrasonography has been widely used in clinical practice in recent years as a noninvasive and rapid examination method, especially for severe pneumonia among adults and children.^10^, ^11^, ^12^ As the chest plain radiograph and CT of COVID‐19 cases show bilateral involvement and peripheral distribution, the pathology is mainly characterized by exudation^1^, ^13^, ^14^, ^15^, ^16^; bedside ultrasonography has played an important role in the diagnosis and treatment of COVID‐19, and our research results have confirmed this point.

The bilateral and unilateral LUSs of the three groups were compared. To reflect the severity of the disease, we chose the most representative highest scores for comparison and found that those of the groups with long‐term residence in Wuhan and with a short‐term stay in Wuhan were significantly different to those of the group that had never visited Wuhan (*z* = −3.720, *p* = .000; *z* = −2.197, *p* = .028); however, there were no differences in the highest scores between the groups with long‐term residence and a short stay in Wuhan (*z* = −1.813, *p* = .070). This shows that lung damage was generally more severe among patients in Wuhan. Some studies also found that the Clinical features of patients in Hubei Province were more serious than those of patients outside Hubei Province. Huang et al.^1^ studied patients in Wuhan and found that all patients had abnormalities on chest CT images. Of the 41 patients, 40 (98%) had bilateral involvement. Xu et al.^17^ studied patients outside Wuhan and found that among 62 patients, all patients except one had abnormalities on chest CT or X‐ray film, and two patients (84%) showed bilateral involvement on chest radiographs. The typical chest CT scan of patients with infection showed bilateral, multilobular, or segmental consolidation areas or bilateral ground‐glass shadows, consistent with the results of the present study. We consider these to be related to viral load; viral transmission occurs only when the viral load reaches a certain quantity, such that the virus has widespread transmissibility, and its transmissibility decreases over time. Some studies estimated the basic reproduction number (R0) of SARS‐CoV‐2 to be 2.24–5.71^18^, ^19^ when it started to spread on December 12, 2019, and the current average estimated value is 2.24–3.58.^18^ Assuming that the epidemic will not recur, R0 is predicted to gradually drop and disappear, similar to SARS.^20^ The rise and decline of viral transmission are related to the genetic mutation of the virus. Angeletti et al.^21^ found that mutations of amino acids in the gene sequence of SARS‐CoV‐2 at a specific site of SARS‐CoV changed its infectivity. A study by Zhang et al.^22^ also found that SARS‐CoV‐2 genetic site mutations were different among patients from different provinces of China.^23^ This mutation can lead to either decreased infectivity or increased infectivity. The repeated prevalence of mutant strains at home and abroad also confirms this point. These factors could have caused the difference in the disease severity between patients in Wuhan and Beijing. Our study also found no significant difference in the incidence and severity between left and right lung disease in patients in either Wuhan or Beijing.

The main limitation of this study was that the participating doctors could not handle all cases at the same time, due to geographical reasons and restricted access to isolation wards. When we compared the severity of the patients in the two different regions, we adopted the highest ultrasound scores. However, during this process, the patients had been treated for different periods of time. Moreover, variations in medical care standards in different regions might have influenced evaluations of ultrasound results. Fortunately, the group with a short‐term stay in Wuhan could be used as a reference in our research to make up for these deficiencies. The statistics of baseline information were rather general, because their changing periods in the development of the disease were not completely synchronized with imaging, and they could not be compared in the same period.

In summary, COVID‐19 showed diffuse lesions in both lungs during disease progression. These lesions mainly surrounded the lungs. The ultrasonographic features of patients in the main affected area of China indicated relatively severe disease. The progression of lung lesions can be visualized using a dynamic monitoring score. Bedside ultrasonography is simple and fast, and therefore plays an important role in the diagnosis of COVID‐19 and can provide timely information for the treatment process.

## AUTHOR CONTRIBUTIONS

Jing Han, Xi Yang, Wei Xu, and Fankun Meng performed the research. Jing Han, Xi Yang, Wei Xu, and Sha Meng designed the research study. Jing Han, Xi Yang, Wei Xu, Ronghua Jin, Sha Meng, Lei Ding, Yuan Zhang, Xing Hu, Weiyuan Liu, and Haowen Li contributed essential reagents or tools. Jing Han, Xi Yang, and Fankun Meng analyzed the data. Jing Han, Xi Yang, and Fankun Meng wrote the paper.

## CONFLICT OF INTEREST

The authors declare no conflict of interest.

## References

[1] Huang C , Wang Y , Li X , et al. Clinical features of patients infected with 2019 novel coronavirus in Wuhan, China. Lancet. 2020;395(10223):497‐506. 10.1016/S0140-6736(20)30183-5 31986264 PMC7159299

[2] World Health Organization. WHO Director‐General's remarks at the media briefing on 2019‐nCoV on 11 Feb 2020. Published online February 11, 2020. Accessed April 28, 2020. https://www.who.int/dg/speeches/detail/who-director-general-s-remarks-at-the-media-briefing-on-2019-ncov-on-11-february-2020

[3] Han Y , Yang H . The transmission and diagnosis of 2019 novel coronavirus infection disease (COVID‐19): a Chinese perspective. J Med Virol. 2020;92(6):639‐644. 10.1002/jmv.25749 32141619 PMC7228390

[4] Caltabeloti FP , Rouby JJ . Lung ultrasound: a useful tool in the weaning process? Rev Bras Ter Intens. 2016;28(1):5‐7. 10.5935/0103-507X.20160002 PMC4828084 27096669

[5] Lichtenstein DA . Current misconceptions in lung ultrasound: a short guide for experts. Chest. 2019;156(1):21‐25. 10.1016/j.chest.2019.02.332 30872018

[6] Picano E , Pellikka PA . Ultrasound of extravascular lung water: a new standard for pulmonary congestion. Eur Heart J. 2016;37(27):2097‐2104. 10.1093/eurheartj/ehw164 27174289 PMC4946750

[7] GOoNH Committee. Office of state administration of traditional Chinese medicine. Notice on the issuance of a programme for the diagnosis and treatment of novel coronavirus (2019‐nCoV) infected pneumonia (trial fifth edition). 2020. Published online February 6, 2020. Accessed April 28, 2020. http://bgs.satcm.gov.cn/zhengcewenjian/2020-02-06/12847.html

[8] Xie X , Zhong Z , Zhao W , Zheng C. , Wang F. , Liu J. Chest CT for typical 2019‐nCoV pneumonia: relationship to negative RT‐PCR testing. Radiology. 2020;296(2):E41‐E45. 10.1148/radiol.2020200343 32049601 PMC7233363

[9] Fang Y , Zhang H , Xie J , et al. Sensitivity of chest CT for COVID‐19: comparison to RT‐PCR. Radiology. 2020;296(2):E115‐E117. 10.1148/radiol.2020200432 32073353 PMC7233365

[10] Pereda MA , Chavez MA , Hooper‐Miele CC , et al. Lung ultrasound for the diagnosis of pneumonia in children: a meta‐analysis. Pediatrics. 2015;135(4):714‐722. 10.1002/ppul.24020 25780071 PMC9923609

[11] Wang Y , Shen Z , Lu X , Zhen Y , Li H . Sensitivity and specificity of ultrasound for the diagnosis of acute pulmonary edema: a systematic review and meta‐analysis. Med Ultrason. 2018;1(1):32‐36. 10.11152/mu-1223 29400365

[12] Rusu DM , Siriopol I , Grigoras I , et al. Lung ultrasound guided fluid management protocol for the critically ill patient: study protocol for a multi‐centre randomized controlled trial. Trials. 2019;20(1):236. 10.1186/s13063-019-3345-0 31023358 PMC6482502

[13] Zhu N , Zhang D , Wang W , et al. A novel coronavirus from patients with pneumonia in China, 2019. N Engl J Med. 2020;382(8):727‐733. 10.1056/NEJMoa2001017 31978945 PMC7092803

[14] Chen N , Zhou M , Dong X , et al. Epidemiological and clinical characteristics of 99 cases of 2019 novel coronavirus pneumonia in Wuhan, China: a descriptive study. Lancet. 2020;395(10223):507‐513. 10.1016/S0140-6736(20)30211-7 32007143 PMC7135076

[15] Chan JF , Yuan S , Kok KH , et al. A familial cluster of pneumonia associated with the 2019 novel coronavirus indicating person‐to‐person transmission: a study of a family cluster. Lancet. 2020;395(10223):514‐523. 10.1016/S0140-6736(20)30154-9 31986261 PMC7159286

[16] Xu X , Yu C , Qu J , et al. Imaging and clinical features of patients with 2019 novel coronavirus SARS‐CoV‐2. Eur J Nucl Med Mol Imaging. 2020;47(5):1275‐1280. 10.1007/s00259-020-04735-9 32107577 PMC7080117

[17] Xu XW , Wu XX , Jiang XG , et al. Clinical findings in a group of patients infected with the 2019 novel coronavirus (SARS‐Cov‐2) outside of Wuhan, China: retrospective case series. BMJ. 2020;368:m606. 10.1136/bmj.m606 32075786 PMC7224340

[18] Zhao S , Lin Q , Ran J , et al. Preliminary estimation of the basic reproduction number of novel coronavirus (2019‐nCoV) in China, from 2019 to 2020: a data‐driven analysis in the early phase of the outbreak. Int J Infect Dis. 2020;92:214‐217. 10.1016/j.ijid.2020.01.050 32007643 PMC7110798

[19] Chen J . Pathogenicity and transmissibility of 2019‐nCoVdA quick overview and comparison with other emerging viruses. Microb Infect. 2020;22(2):69‐71. 10.1016/j.micinf.2020.01.004 PMC7102641 32032682

[20] Thompson RN . Novel coronavirus outbreak in Wuhan, China, 2020: intense surveillance is vital for preventing sustained transmission in new locations. J Clin Med. 2020;9(2):498. 10.3390/jcm9020498 PMC7073840 32054124

[21] Angeletti S , Benvenuto D , Bianchi M , Giovanetti M. , Pascarella S. , Ciccozzi M. COVID‐2019: the role of the nsp2 and nsp3 in its pathogenesis. J Med Virol. 2020;92(6):584‐588. 10.1002/jmv.25719 32083328 PMC7228367

[22] Zhang L , Shen FM , Chen F , Lin Z . Origin and evolution of the 2019 novel coronavirus. Clin Infect Dis. 2020;71(15):882‐883. 10.1093/cid/ciaa112 32011673 PMC7108176

[23] Wu D , Zou S , Bai T , et al. Poultry farms as a source of avian influenza A (H7N9) virus reassortment and human infection. Sci Rep. 2015;5:7630. 10.1038/srep07630 25591105 PMC4295517

